# The changing landscape of nontyphoidal salmonellosis: epidemiological patterns, imported cases and serovar distribution in Germany from 2012 to 2023

**DOI:** 10.1186/s12879-025-10907-5

**Published:** 2025-04-10

**Authors:** Simon Brinkwirth, Achim Dörre, Klaus Stark, Anika Meinen

**Affiliations:** 1 Department of Infectious Disease Epidemiology, Robert Koch Institute, Zoonoses and Tropical Infections, Seestr. 10, 13353 Berlin, Germany; 2 Postgraduate Training for Applied Epidemiology (PAE), Robert Koch-Institute, Berlin, Germany; 3 European Programme for Intervention Epidemiology Training (EPIET), European Centre for Disease Prevention and Control (ECDC), Stockholm, Sweden; 4 Department of Infectious Disease Epidemiology, Focal Point for the Public Health Service, Crisis Management, Outbreak Investigations and Training Programmes, Robert Koch Institute, Seestr. 10, 13353 Berlin, Germany

**Keywords:** Salmonellosis, Epidemiology, Serovar, Imported cases, Germany, Incidence trends

## Abstract

**Introduction:**

Nontyphoidal *Salmonella* is a zoonotic foodborne pathogen that represents a global public health issue. In the European Union and Economic Area, about 66,000 cases of reported nonthyphoidal salmonellosis occurred in 2022, with about 9,100 cases in Germany. The aim of this study is to analyse the incidence and epidemiological characteristics as well as trends of salmonellosis in Germany from 2012 to 2023.

**Methods:**

German national surveillance data on salmonellosis from 2012 to 2023 were analysed. Available information included demographics, notification dates, country of exposure, hospitalisation, and serovar. The incidence was calculated per 100,000 population, stratified by age, sex, and travel and hospitalisation history. A descriptive analysis was conducted.

**Results:**

A total of 160,782 cases of salmonellosis were reported between 2012 and 2023 in Germany, with seasonal peaks occurring during the summer months. The incidence declined from 26 per 100,000 in 2012 to 13 per 100,000 in 2023. This decline was observed across all defined age groups, sex and regions. The proportion of imported cases increased since 2012, reaching a peak of 26% (*n* = 1,943) in 2023. The proportion of cases that resulted in hospitalisation remained relatively constant, accounting for approximately 30% of all cases. The incidence was higher in males and children under the age of five years. The most frequent serovars were *S*. Enteritidis and *S*. Typhimurium. From 2020 onwards, there was an increase in the number of unknown serovars.

**Conclusion:**

The analysis of these surveillance data provided a good basis to monitor trends and to identify special population groups at risk. The decrease in the incidence of salmonellosis in Germany between 2012 and 2023 might reflect a positive trend in public health efforts and food safety. The increased proportion of imported cases highlights the higher importance of monitoring and addressing travel-related exposures. Ongoing efforts are essential to mitigate both domestic and imported salmonellosis cases, particularly in young children and older adults.

**Supplementary Information:**

The online version contains supplementary material available at 10.1186/s12879-025-10907-5.

## Introduction

Nontyphoidal *Salmonella* is one of the most prevalent zoonotic foodborne pathogens and a global public health concern, responsible for the development of salmonellosis (hereafter referred to as salmonellosis) [1]. In the European Union and Economic Area (EU/EEA), about 66,000 cases of salmonellosis were reported in 2022, the second most frequently reported bacterial gastrointestinal infection in humans after campylobacteriosis [2]. The total economic burden of salmonellosis in humans is estimated to be up to 3 billion euros per year by the European Food Safety Authority (EFSA) [3]. In Germany, as in the EU/EEA, salmonellosis is the second most frequent bacterial gastrointestinal disease, with about 9,100 annual cases in 2022 [4].

Since its first isolation in 1884, S*almonella* has been divided into two different species, six subspecies and over 2,500 different serovars [5]. In human infections, salmonellosis is mainly caused by nontyphoidal species *Salmonella enterica* and therefore of particular importance for surveillance. *Salmonella* spp. are transmitted by eating contaminated food (e.g., eggs and raw meat) or via the fecal-oral route [6, 7]. Common symptoms of salmonellosis are fever, diarrhea, and abdominal pain. In rare cases, salmonellosis can lead to septicemia and even death. The incubation period is six hours to three days, depending on the dose and the immune status of the host [8]. Very young children and those with compromised immune systems are at the highest risk of invasive infections and hospitalisation due to salmonellosis [9].

To address the high prevalence of salmonellosis in the early 2000s, the EU passed regulations for the poultry, swine, and cattle industries. The regulations were for the detection, control, and poultry vaccination measures for *Salmonella* spp. at all stages of food production. Primary production and animal feed were key target areas to reduce the prevalence of these pathogens and the associated public health risk [10, 11]. These coordinated efforts have led to a decrease of salmonellosis cases in the EU by nearly 50% from 2004 to 2009 and a continuing decline thereafter [3].

Despite these efforts, the consumption of (undercooked) eggs and meat in Germany, and travelling outside Germany, remain risk factors for salmonellosis [12]. However, the number of travellers in 2024 reached the highest level in human history, with 1.5 billion arrivals worldwide compared to only half that number in 2005 [13]. This increase in travelling could have an impact on the distribution of *Salmonella* serovars.

The existing literature does not provide a detailed understanding of the long-term epidemiological trends; in particular, there is a lack of knowledge about the long-term variations of different *Salmonella* serovars and the epidemiological characteristics of imported cases. Given the overall decline in salmonellosis cases since 2001, it is of public health importance to assess possible opposing trends, e.g. in specific demographic subgroups.

In this study, we analyse the incidence of salmonellosis and epidemiological characteristics in Germany from 2012 to 2023, based on national surveillance data, along the following research questions:

(A) How did the incidence and epidemiological patterns of salmonellosis vary over time in terms of demographics, seasonality, and serovar distribution?
(B) What was the proportion of imported cases and how did it change over time?

We aim to provide a thorough understanding of the multifaceted aspects of salmonellosis in Germany and to provide evidence for public health action.

## Methods

In Germany, the detection of *Salmonella* in humans is notifiable according to the Protection against Infections Act (Infektionsschutzgesetz; IfSG). In this study, we analysed routine national surveillance data on salmonellosis cases from 1 January 2012 to 31 December 2023 (data status as of 1 March 2024), including age, sex, place of residence, date of notification, country of exposure, hospitalisation and serovar detected (Table 1).

Our data analysis focused exclusively on salmonellosis cases defined according to the Robert Koch Institute (RKI) reference definition,

1. Salmonellosis case - reference definition: Individuals must exhibit at least one clinical symptom associated with salmonellosis, such as diarrhea, abdominal pain, fever, or vomiting. In addition, a definitive laboratory-confirmed diagnosis of a *Salmonella* infection or a compelling epidemiological confirmation establishing a direct linkage to a laboratory-confirmed person or a specific contaminated food item is required.
2. Imported Case: A case exposed outside of Germany.
3. Hospitalised Case: A case hospitalised due to salmonellosis. Valid data on the hospitalisation was available from 2017.
4. Serovars: Detailed analysis includes *Salmonella enterica* subsp. *enterica* (*S*.) Typhimurium (including the monophasic variant), *Salmonella enterica* subsp. *enterica* (*S*.) Enteritidis, *Salmonella enterica* subsp. *enterica* (*S*.) Infantis, *Salmonella enterica* subsp. *enterica* (*S*.) Derby, and *Salmonella enterica* subsp. *enterica* (*S*.) Muenchen. The category *Other* combines serovars other than these. *Unknown* means that the serovar was not identified or not reported by the laboratory including those where the information was only available at group level but not at serovar level.
5. Population: Defined according to the German population in the years 2012–2023 [14].
6. Region of the notification (federal states): North: Bremen, Hamburg, Lower Saxony, Schleswig-Holstein, Mecklenburg-Western Pomerania; East: Saxony, Thuringia, Berlin, Brandenburg, Saxony-Anhalt; South: Baden-Württemberg, Bavaria; West: North Rhine-Westphalia, Hesse, Rhineland-Palatinate, Saarland.

The IfSG regulates infectious disease surveillance in Germany. Physicians and laboratories report specific infectious diseases or pathogen detections to local public health departments, which investigate and respond to these cases. Local departments send pseudonymised notifications that meet German case definitions to regional public health departments, which then forward them to the Robert Koch Institute, the national public health institute [15].

### Data analysis

**Fig. 1:**
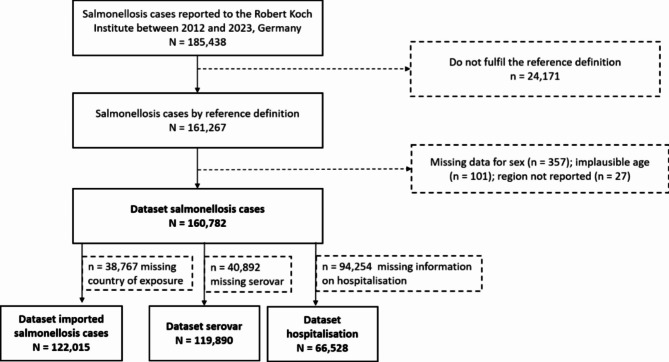
Study flow chart of included reported salmonellosis cases in Germany, 2012–2023

We performed a descriptive analysis to assess the incidence and proportion on epidemiological patterns of reported salmonellosis cases in Germany from 2012 to 2023. The dataset consists of all salmonellosis cases reported to the Robert Koch Institute according to the IfSG. Cases with missing information on sex or region as well as implausible entries for age were excluded (Fig. 1). The cleaned dataset was used for further analysis. Age groups were divided into five year intervals from 0–4 years to 75–79 years, and all persons aged 80+.

The incidence of salmonellosis has been determined as the number of reported cases per 100,000 population– for the whole population and separately for age group, sex, and imported cases. Population data were available from the Federal Statistical Office. Descriptive analysis was performed based on all salmonellosis cases and further stratified by age and sex. Sub-analyses was performed for imported cases, hospitalised cases and those with available information regarding the serovar. The dataset was analysed with R version 4.2.1 using the R packages ggplot2 and gtsummary.

## Results

### Overview of salmonellosis notifications and data completeness

A total of 160,782 salmonellosis cases were reported in Germany between 2012 and 2023. For 119,890 cases (75%) the serovar of *Salmonella* spp. was available with a decreasing proportion to 61% in 2023. For 40,892 cases (25%), a specific serovar was not notified. For these cases the serovar was classified as unknown. Information on the country of exposure was available for 122,015 cases (76%), allowing the classification of imported cases. Between 2012 and 2016, the quality of data on hospitalisation was inadequate due to the introduction of a new variable into the surveillance system in 2011. We therefore analysed the data regarding hospitalisation from 2017 onwards, with available information on hospitalisation due to salmonellosis for 66,528 cases (85% for the years 2017–2023). A more detailed distribution for each year is shown in Table 1.

**Table 1:** Summary table: salmonellosis cases in Germany from 2012 to 2023

Characteristics	Total (%)	2012	2013	2014	2015	2016	2017	2018	2019	2020	2021	2022	2023
Cases	160,782	20,836	18,956	16,221	13,768	12,941	14,208	13,491	13,703	8,684	8,191	9,117	10,666
*Sex*													
Female	78,084 (49)	10,107 (49)	9,243 (49)	7,913 (49)	6,736 (49)	6,364 (49)	6,900 (49)	6,648 (49)	6,615 (48)	4,208 (48)	3,949 (48)	4,321 (47)	5,080 (48)
Male	82,698 (51)	10,729 (51)	9,713 (51)	8,308 (51)	7,032 (51)	6,577 (51)	7,308 (51)	6,843 (51)	7,088 (52)	4,476 (52)	4,242 (52)	4,796 (53)	5,586 (52)
*Serovar*													
*Information available*	*119*,*890*	*16*,*855*	*14*,*901*	*12*,*602*	*10*,*281*	*9*,*819*	*10*,*860*	*10*,*176*	*10*,*001*	*6*,*089*	*5*,*616*	*6*,*223*	*6*,*467*
*S*. Typhimurium	43,906 (37)	6,869 (41)	6,126 (40)	5,098 (41)	3,864 (38)	3,562 (36)	3,709 (34)	3,380 (33)	3,336 (33)	2,222 (37)	1,999 (36)	1,852 (30)	1,889 (29)
*S*. Enteritidis	46,952 (39)	6,477 (38)	5,152 (34)	4,410 (35)	4,095 (40)	4,036 (41)	4,685 (43)	4,576 (45)	4,145 (42)	2,218 (36)	2,117 (38)	2,324 (37)	2,717 (42)
*S.* Infantis	3,504 (2.9)	334 (2)	685 (4.5)	333 (2.6)	250 (2.4)	284 (2.9)	269 (2.5)	269 (2.6)	349 (3.5)	230 (3.8)	155 (2.8)	207 (3.3)	139 (2.2)
*S.* Derby	1,760 (1.5)	208 (1.2)	233 (1.5)	267 (2.1)	127 (1.2)	154 (1.6)	114 (1)	156 (1.5)	148 (1.5)	106 (1.7)	95 (1.7)	69 (1.1)	83 (1.3)
*S.* Muenchen	844 (0.7)	66 (0.4)	224 (1.5)	226 (1.8)	23 (0.2)	32 (0.3)	22 (0.2)	31 (0.3)	25 (0.3)	133 (2.2)	27 (0.5)	22 (0.4)	13 (0.2)
*Other*	22,924 (19)	2,901 (17)	2,481 (16)	2,268 (18)	1,922 (19)	1,751 (18)	2,061 (19)	1,764 (17)	1,998 (20)	1,180 (19)	1,223 (22)	1,749 (28)	1,626 (25)
*Countries of exposure*													
Information available	122,015	19,509	16,441	12,581	10,449	9,175	9,780	9,389	9,676	5,614	5,532	6,376	7,493
Country other than Germany	20,045 (16)	1,908 (10)	1,892 (12)	1,848 (15)	1,857 (18)	1,795 (20)	2,116 (22)	2,161 (23)	2,289 (24)	383 (7)	522 (9)	1,331 (21)	1,943 (26)
*Hospitalisation*													
Information available	66,528	-	-	-	-	-	12,098	11,670	11,960	7,341	7,013	7,567	8,879
Hospitalised	20,172 (30)	-	-	-	-	-	3,731 (31)	3,392 (29)	3,640 (30)	2,221 (30)	2,200 (31)	2,293 (30)	2,695 (30)

### Salmonellosis incidence

**Fig. 2:**
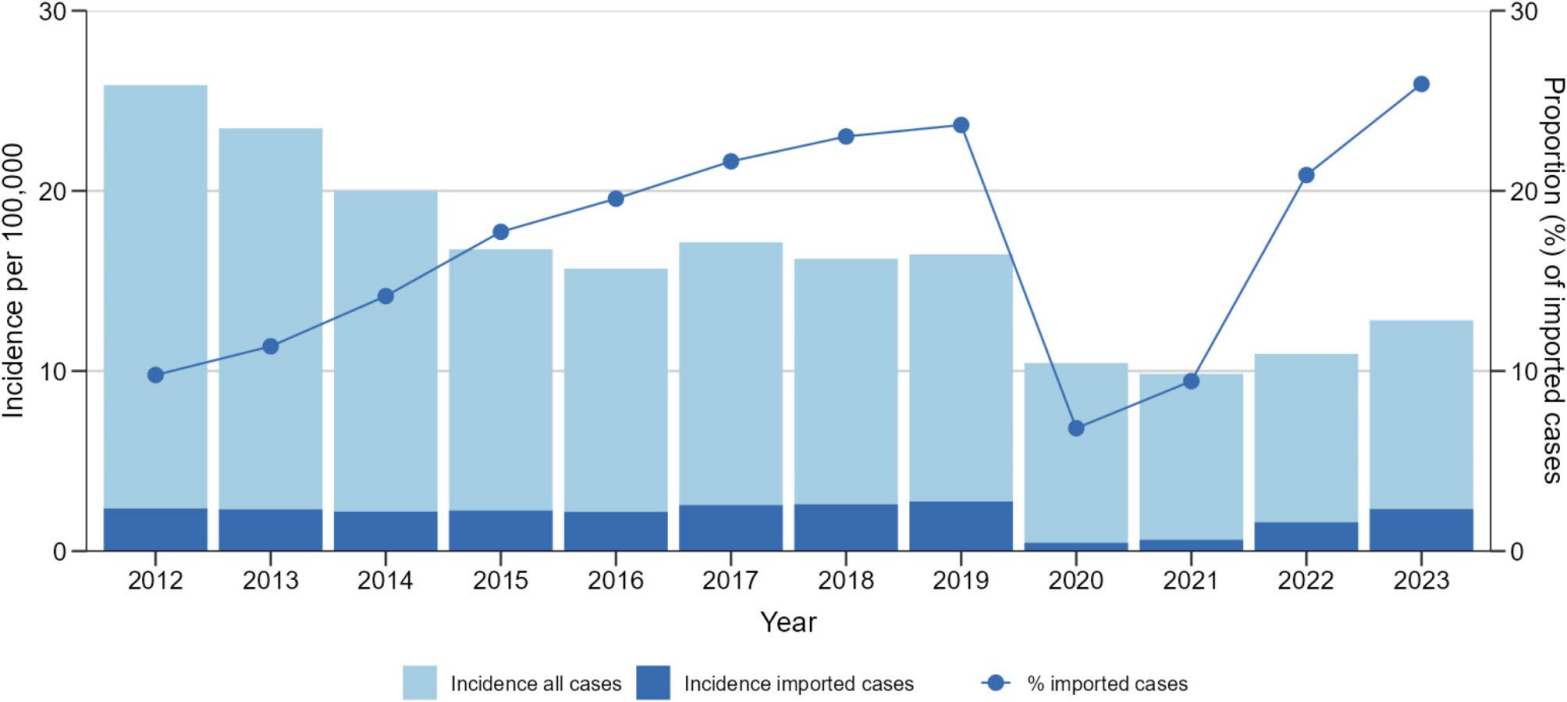
Salmonellosis incidence per 100,000 population with proportion of imported cases in Germany from 2012–2023

The annual incidence (cases per 100,000 population) decreased from 26 (*n* = 20,836) in 2012 to 13 (*n* = 10,666) in 2023 (Fig. 2). Before the SARS-CoV-2 pandemic, the annual incidence decreased between 2012 and 2015 and stabilised at a plateau between 2015 and 2019, ranging between 16 and 17 cases per 100,000 population. In 2020, the incidence declined by about 30% and showed a steady increase in the following years, but did not reach the pre-pandemic levels of the plateau phase. This trend was observed in all geographic regions, although there was an uneven distribution of the total incidence, with the highest incidence by region in East Germany (Supplementary Figure S1).

### Proportion and trends of imported cases

Of all cases with available information, 20,045 (16%) were classified as imported cases. The proportion of imported cases increased from 10% in 2012 to 24% in 2019, showed a sharp decline in the pandemic years 2020 and 2021, and peaked at 26% in 2023 (Fig. 2). Among the imported cases, the most frequently reported countries of exposure were Türkiye (20%; *n* = 4,002), Egypt (11%; *n* = 2,283), Thailand (7%; *n* = 1,327), and Spain (6%; *n* = 1,229). In 84% of cases, the country of exposure was Germany (90,311 cases).

### Proportion of hospitalisation (2017–2023)

Overall, 20,172 (30%) of the total cases were reported as hospitalised, with the proportion of hospitalised cases remaining stable between 2017 and 2023, ranging from 29 to 31%.

### Demographic characteristics

**Fig. 3:**
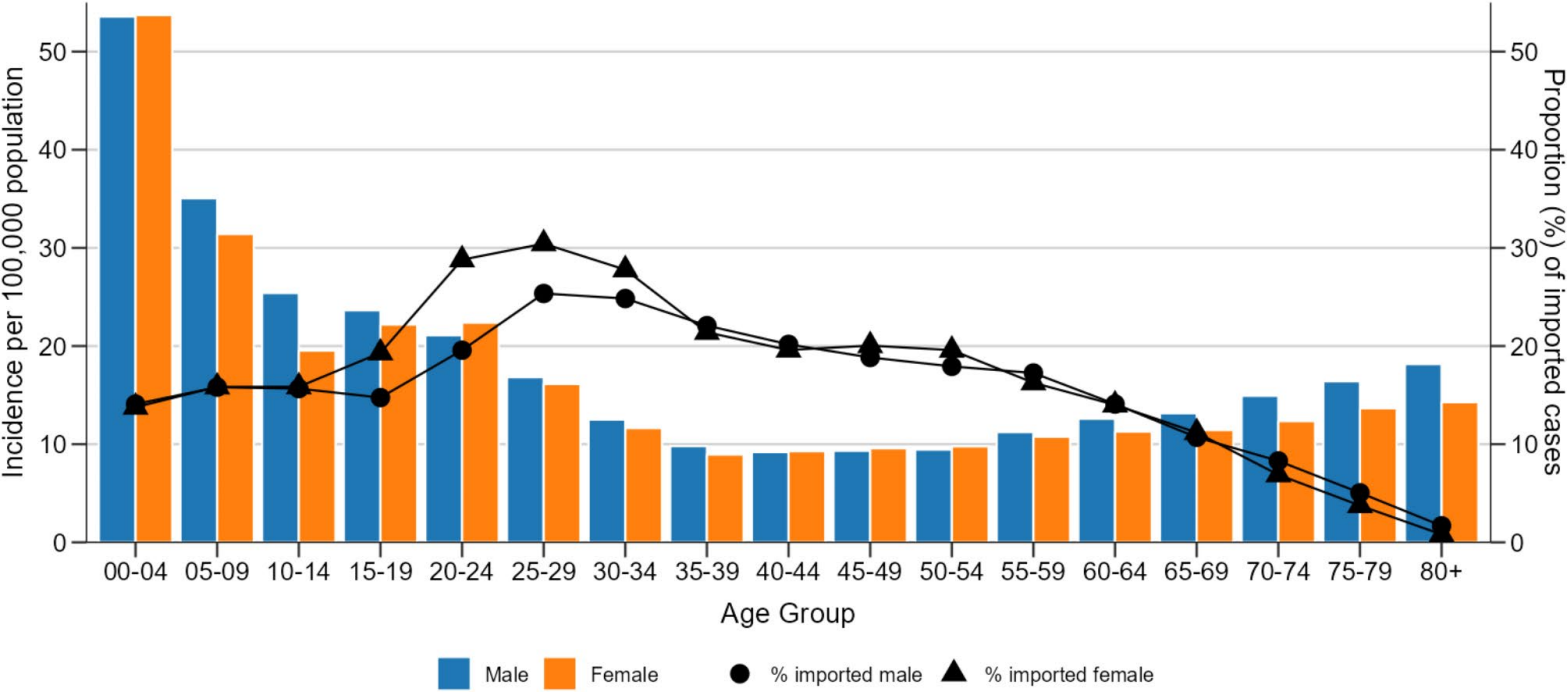
Salmonellosis incidence per 100,000 by age and sex with proportion of imported cases in Germany from 2012–2023

The mean incidence of salmonellosis was slightly higher in males (18 cases per 100,000) than in females (17 cases per 100,000). In most age groups, males were more affected than females or the incidences were similar. One exception were the 20–24-year-olds where females were more affected than males. The highest incidence was observed in children under five years old, with 54 cases per 100,000, followed by the 5–9-year-olds with 33 cases per 100,000. The incidence decreased steadily until the age group 40–44 years, then increased, peaking in those aged 80+ years at 16 cases per 100,000 (Fig. 3). The overall decreasing trend of salmonellosis over the years is observed across all age groups (Supplementary Figure S2).

### Demographic distribution of imported cases

For imported cases in total, females and males were affected similarly (2.2 *vs.* 2.1 cases per 100,000, respectively). The proportion of imported cases varied between age groups and sex, with females, particularly those aged 25–29 years, showing the highest proportion of imported cases (up to 30%) (Fig. 3).

### Hospitalisation by age and sex

Among hospitalised cases, males had a higher incidence across most age groups. Notably, age groups under 20 years and those over 60 years exhibited a higher proportion of hospitalised cases, with a pronounced difference in hospitalisation proportion between males and females (Supplementary Figure S3).

### Seasonality

**Fig. 4:**
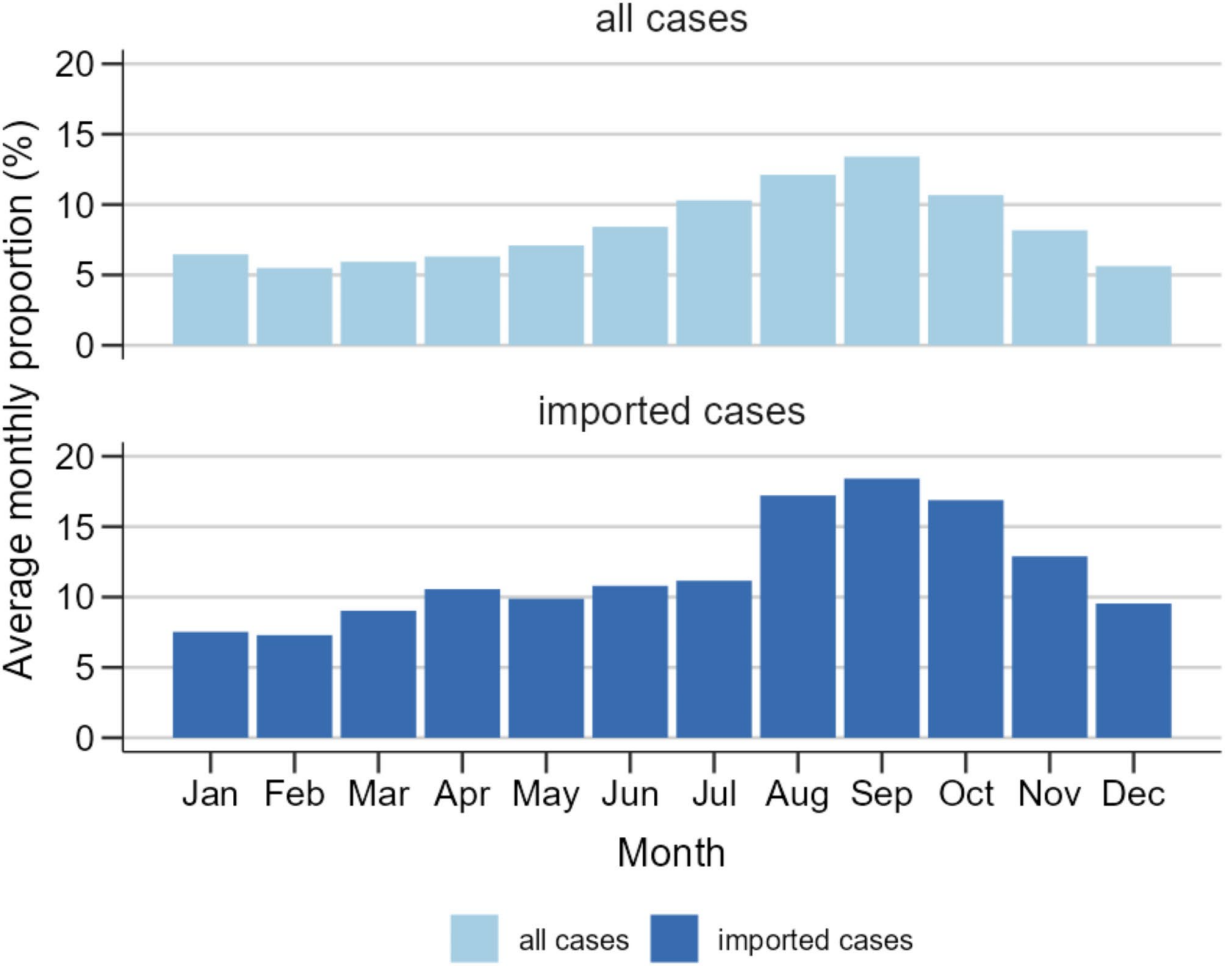
Seasonal average monthly proportion of salmonellosis cases and average monthly proportion of imported cases from 2012–2023 in Germany

The number of reported salmonellosis cases remained relatively low and stable from January to April, with a marked increase from May onwards, peaking in September (Fig. 4). A smaller secondary peak was observed in January. This seasonal pattern was consistent over the various years considered and for all age groups, including all cases and those defined as imported. In September, the average proportion of imported cases among all cases is highest (18.4%) (Fig. 4). The proportion of cases hospitalised remained stable throughout the year, ranging from 28 to 33%.

### Serovar distribution and trends

**Fig. 5:**
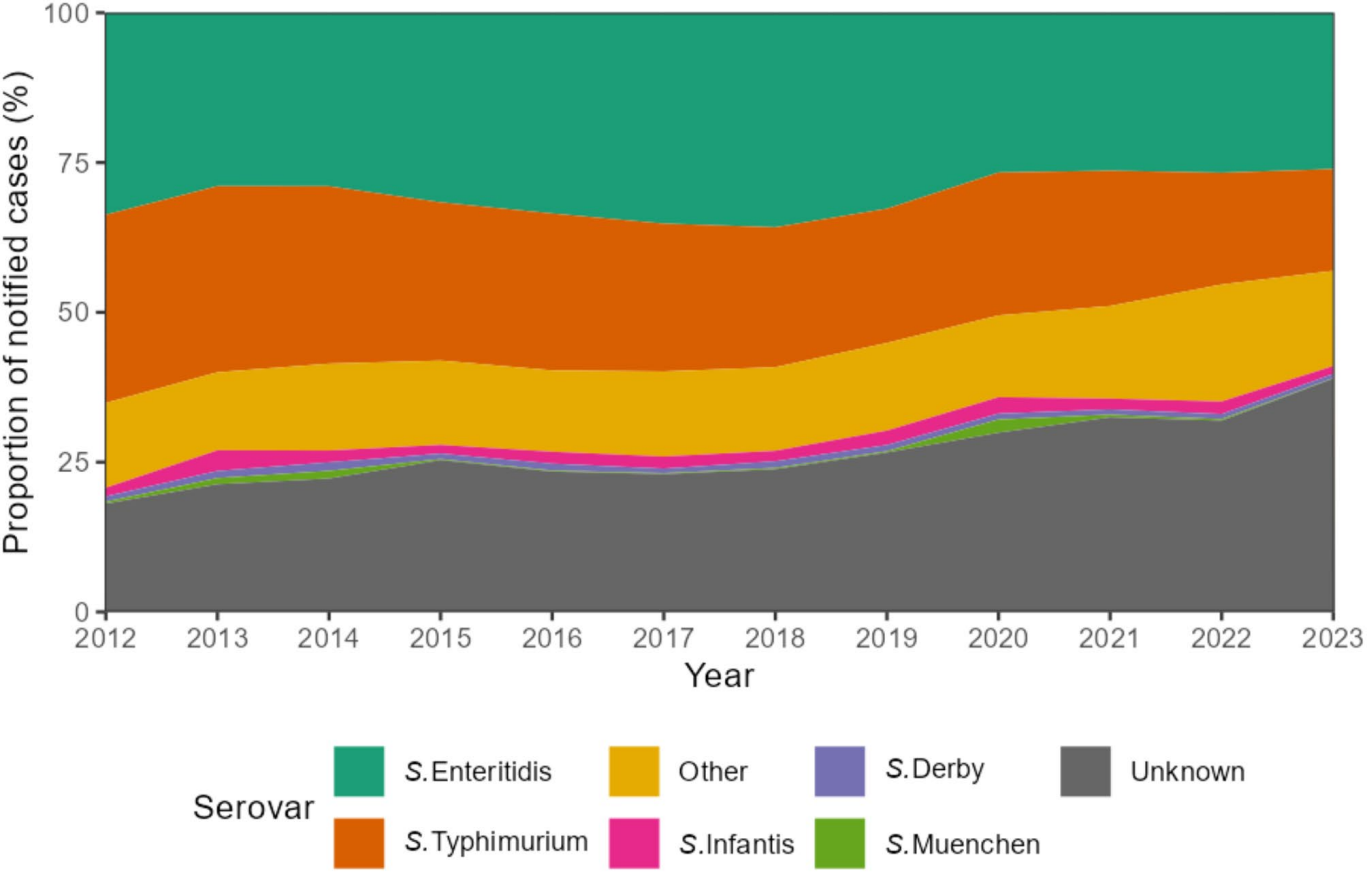
Serovar distribution in salmonellosis cases from 2012–2023 in Germany

The number of cases with unknown serovars increased from 2,595 in 2020 to 4,199 in 2023 (Fig. 5). The following data relate only to the cases where serovar information was available. Among all cases where serovar information was available, the most frequently identified serovars were *S.* Enteritidis (39%; *n* = 46,952) and *S.* Typhimurium (37%; *n* = 43,906). Other important serovars included *S.* Infantis (2%; *n* = 3,504), *S.* Derby (1%; *n* = 1,760), and *S.* Muenchen (0.5%; *n* = 844). The proportion of *S*. Enteritidis varied over the years, with a minimum of 35% of cases in 2014 and a maximum of 45% in 2018. The proportion of *S.* Typhimurium remained consistent until 2019 but exhibited a decline in recent years, from a maximum of 41% in 2012 to a minimum of 29% in 2023. The proportions of less frequent serovars remained relatively stable.

### Serovar distribution in imported cases

The overall serovar distribution in imported cases followed a similar pattern as in the general population, though with a larger difference between *S*. Typhimurium (36%; *n* = 33,021) and *S*. Enteritidis (40%; *n* = 36,407) (Supplementary Figure S4).

## Discussion

The data showed a substantial decrease in the incidence of salmonellosis across Germany; observed in all age groups, sex, and regions. Notably, the strong decrease in incidence in 2020 coincides with the SARS-CoV-2 pandemic. Despite the overall decrease, the proportion of imported cases increased, reaching 26% in 2023.

The observed incidence trend is in line with the overall dynamics of salmonellosis in the EU/EEA. After a substantial decrease in cases in 2020 coinciding with the SARS-CoV-2 pandemic, the number of cases increased again from 2022 onwards, but still remained below the pre-pandemic levels as observed in other countries [16]. The travel and contact restrictions and possible changes in eating habits during the pandemic may have led to the lower salmonellosis incidence [17]. In addition, the possibility of a surveillance artefact due to health system congestion and social impact and reluctance of the population to seek medical care must be considered. However, even during the pandemic years, the proportion of hospitalised cases remained constant, suggesting that a comparable proportion of the population was covered by the surveillance system and not specific subgroups of e.g. severe cases.

The incidence in Germany is lower than the EU/EEA average observed over the years [2]. Differences in incidence may be due to a number of reasons, and exact figures should be compared with caution as there are differences in salmonellosis surveillance systems and specific case definitions across the EU. The case definition in Germany was changed in August 2022 to also include a positive PCR test as a confirmed case; previously, cultural evidence was required [18]. In our study, potential changes in incidence due to the change in case definition would only have been visible from 2023 onwards. However, it is not possible to assess the potential impact at this stage.

The higher incidence among males and certain age groups, particularly children under five years old, and adults over 70, underline the vulnerability of these groups and are consistent with previous studies and global surveillance data [19, 20]. Similarly, the higher hospitalisation in young children and the elderly emphasise the need for careful monitoring and prevention in these populations.

The number of imported cases in our data and the increasing importance of travel for salmonellosis in Germany is consistent with a global meta-analysis of risk factors for sporadic salmonellosis, which identified travel as one of the main risk factors, with travel abroad posing a higher risk than travel within a country [12]. However, in some countries, such as Iceland, Finland, Norway and the United Kingdom, travel-associated infections have a much higher impact, accounting for about 40–60% of national cases, compared to Germany with about 25% [2, 21]. This may be explained by differences in travel destination or differing risks for (food-borne) autochthonous infections. The extent of travel-related cases should therefore continue to be monitored. When considering the most common exposure country, we should always take the high number of travellers to certain countries into account to correctly interpret the different risks [22]. Since Türkiye and Spain are among the most common destinations for travellers from Germany, they should not generally be interpreted as countries with a high risk of salmonellosis. However, countries like Egypt and Thailand account for a higher proportion of the total imported salmonellosis cases, even though they are much less common destinations for German travellers [23].

Salmonellosis has a stable seasonality over the years of surveillance. Possible explanations for the high incidences during the summer months could be higher bacterial loads in food, certain practices, where food hygiene is more difficult to apply (e.g. during barbeques) or changes in food consumption habits (e.g. consumption of more uncooked foods like salads or fruits). In the context of the changing climate, initial studies have focused on the potential impact of heat waves on some *Salmonella* serovars. This may play a greater role in the near future, with public health interventions needed before and during heatwaves, as recommended by Milazzo et al. [24]. The consistent proportion of hospitalised cases observed throughout the year suggests that the severity of the pathogens remains relatively constant, while the total number of infections exhibits seasonal fluctuations.

The most frequently identified serovars in Germany, in Europe and worldwide over the years have been *S*. Enteritidis and *S*. Typhimurium [2, 25]. These serovars remained most prevalent even after the EU regulations for the detection, control, and poultry vaccination in the early 2000s [10, 11]. A meta-analysis focusing on serovars in animal-based foods reflects the high prevalence of *S*. Enteritidis in Europe, which is most prevalent in poultry, and *S.* Typhimurium, which is most prevalent in pork, beef and seafood [26]. Compared to the EU average, Germany has a higher prevalence of *S*. Typhimurium, which is partly due to the importance of pork in the diet (e.g., in some regions consumption of pork including raw minced pork is particularly popular). However, total meat consumption decreased from 61.5 kg/capita in 2012 to 51.6 kg/capita in 2023 in Germany, possibly contributing to the overall decrease in salmonellosis cases [27]. This is consistent with the decrease of *S*. Typhimurium cases in recent years. Outbreaks in certain years, especially of less common serovars, can cause a sudden increase followed by a decline and disappearance in the serovar distribution, such as *S*. Muenchen in 2013 and 2014 in Germany [28].

The sharp increase in unknown serovars from 30% in 2020 to over 39% in 2023 might be due to a shift to PCR-based methods. Using solely this diagnostic methods, it is not possible to differentiate between serovars. A survey among diagnostic laboratories in Germany in 2024 supports this hypothesis revealing that some of them already replaced cultural by culture-independent methods (not published). Previous studies have shown that culture-independent tests for *Salmonella* offer opportunities for timely diagnosis with reduced costs, but also new challenges [29]. Cultivation of isolates is essential for sequencing and the implementation of genomic surveillance enabling a better detection of outbreaks.

### Limitations

This study analyses national surveillance data on salmonellosis in Germany and describes the epidemiological patterns and trends of salmonellosis cases over more than a decade of surveillance. However, there are limitations: Due to the nature of salmonellosis surveillance, reported cases are likely not representative of all salmonellosis infections in the population. The surveillance data include individuals residing in Germany and accessing the healthcare system. Various estimations of the level of under-reporting and underdetection of salmonellosis infections suggest that the true observed number of cases is two to ten times higher than the reported numbers. Reasons for under-reporting range from non-use of medical care, especially for those with mild infections, to the absence of diagnostic tests for every patient with diarrhoeal illness [30].

Reporting biases regarding the definition of imported cases or their allocation to a certain destination could occur when cases visited more than one country or already returned to Germany within the possible incubation period and might have acquired the infection there. This could either result in under- or overestimation of imported cases or certain destinations. Moreover, food consumption patterns may vary according to different travel destinations.

Based on the surveillance data alone it is not possible to explain the rise in unknown serovars, although some data indicate that a shift towards culture-independent methods in the diagnostics could be one plausible explanation. We presented the distribution of serovars among those cases with known serovar. However, we do not know if this distribution of serovars is similar to those where this information is missing.

Our results and the quality of the data depend on over 400 local health authorities reporting salmonellosis to state and federal authorities. Due to the nature of surveillance data and varying allocation of resources, it is not possible to ensure the same data quality across all health authorities. The introduction of new variables in the reporting data format may require a significant investment of time and resources. For Germany, data on the hospitalisation due to *Salmonella* was established in 2011, but only valid from 2017 onwards, as the previous format lacked the necessary accuracy.

In addition, the SARS-CoV-2 pandemic occurred during our observation period, which may have affected the incidence of salmonellosis and other infectious diseases due to changes in health-seeking and diagnostic behaviour [31]. Our results are consistent with European trends of decreasing salmonellosis incidence, which may reflect improved food safety and hygiene practices.

## Conclusion

Surveillance data provide a good data basis to monitor trends and to identify special population groups at risk. The decrease in the incidence of salmonellosis in Germany between 2012 and 2023 might reflect a positive trend in public health efforts and food safety. The proportion of imported cases has increased, highlighting the higher importance of monitoring travel-related exposures and addressing them by targeted pre-travel advice. We identified both children under five and older adults having a higher risk for severe infections showing the need for targeted prevention strategies in these age groups. Infections peaking in the summer months could be a signal for the need to increase public awareness of food hygiene during this period. We identified an increasing lack of serovar identification over the years for which changes in diagnostic methods may be an explanation. In summary, we were able to detect trends, groups at risk as well as data gaps which will potentially complicate outbreak detection emphasising the importance of continued surveillance in order to enable the responsible authorities to adapt public health strategies effectively and timely.

## Electronic supplementary material

Below is the link to the electronic supplementary material.

Supplementary Material 1

## Data Availability

The datasets generated and/or analysed during the current study are not publicly available due to to privacy/ethical restrictions but are available from the corresponding author on reasonable request.

## Abbreviations

EU/EEA: European Union and European Economic Area.
EFSA: European Food Safety Authority.
IfSG: Protection against Infections Act (German: Infektionsschutzgesetz).
PCR: Polymerase Chain Reaction.
RKI: Robert Koch Institute (national public health institute).
