# Supplementary material for: The changing landscape of nontyphoidal salmonellosis: epidemiological patterns, imported cases and serovar distribution in Germany from 2012 to 2023
Source: BMC Infect Dis. 2025 Apr 10;25:497. doi: 10.1186/s12879-025-10907-5 (PMC11984224; doi:10.1186/s12879-025-10907-5)
Supplement: Supplementary file 1 — Supplementary Material 1 [file 12879_2025_10907_MOESM1_ESM.docx]

Supplement: The Changing Landscape of Nontyphoidal Salmonellosis: Epidemiological Patterns, Imported Cases and Serovar Distribution in Germany from 2012 to 2023

Simon Brinkwirth^1,2,3^, Achim Dörre^4^, Klaus Stark^1^, Anika Meinen^1*^

^1^Robert Koch Institute, Department of Infectious Disease Epidemiology, Unit 35: Gastrointestinal Infections, Zoonoses and Tropical Infections, Seestr. 10, 13353 Berlin, Germany

^2^Postgraduate Training for Applied Epidemiology (PAE), Robert Koch-Institute, Berlin, Germany

^3^European Programme for Intervention Epidemiology Training (EPIET), European Centre for Disease Prevention and Control (ECDC), Stockholm, Sweden.

^4^Robert Koch Institute, Department of Infectious Disease Epidemiology, Unit 31: Focal Point for the Public Health Service, Crisis Management, Outbreak Investigations and Training Programmes, Seestr. 10, 13353 Berlin, Germany

*Corresponding author: Anika Meinen

Email: meinena@rki.de

Phone: (+49) 3018754-3663

Keywords:

Salmonellosis; Epidemiology; Serovar; Imported Cases; Germany; Incidence Trends

Supplemental Figures

An overall increasing trend for nontyphoidal salmonellosis is observed across all German regions, nevertheless there is an unequal distribution of the total incidence of salmonellosis within the observed period. The *East* is identified as a high-incidence area with a mean incidence of 24 cases per 100,000 inhabitants compared to the *North* (15 cases), *South* (14 cases) and *West* (15 cases).


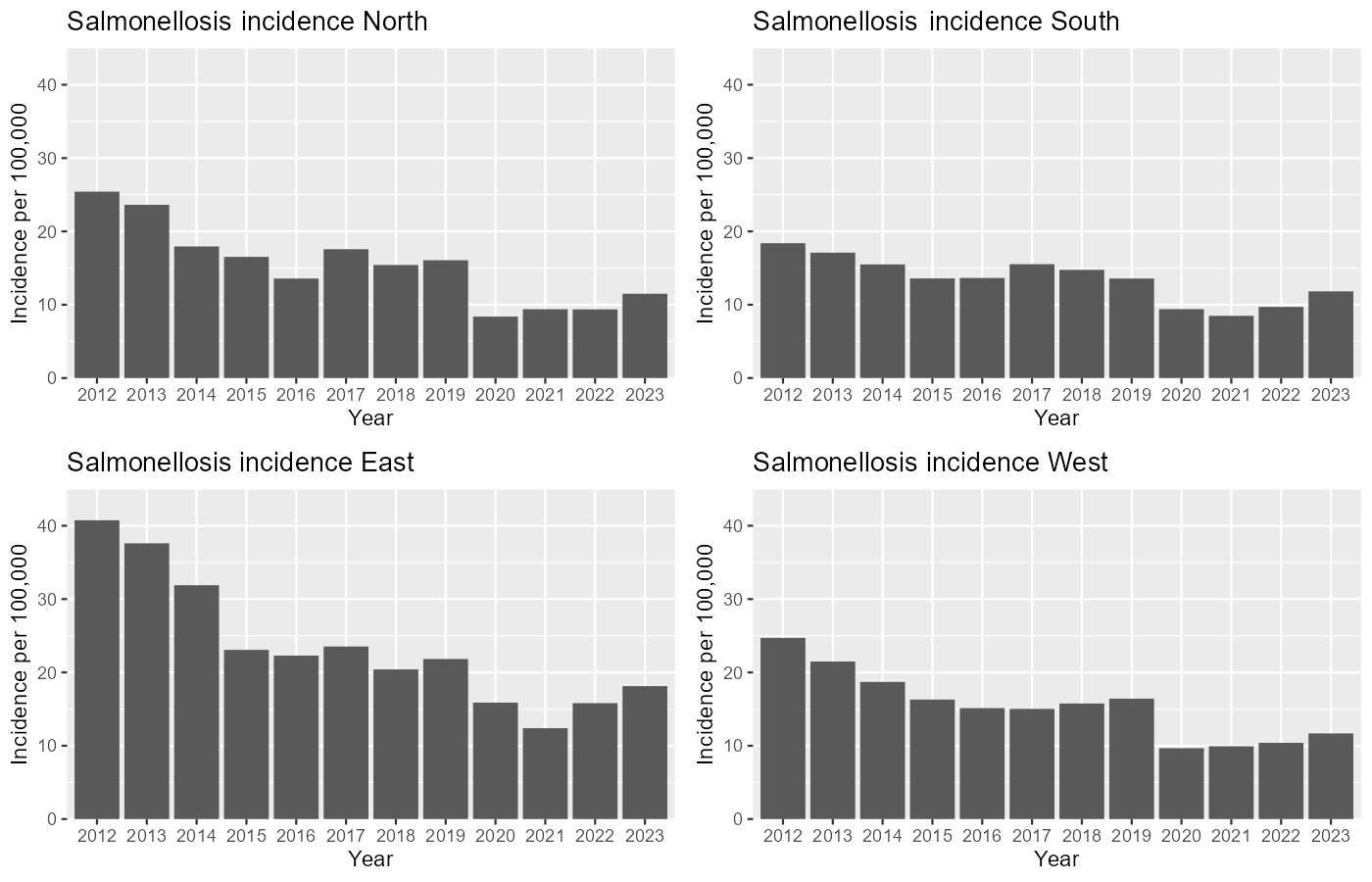


Figure S1. Salmonellosis incidence per 100,000 population from 2012–2023 per region


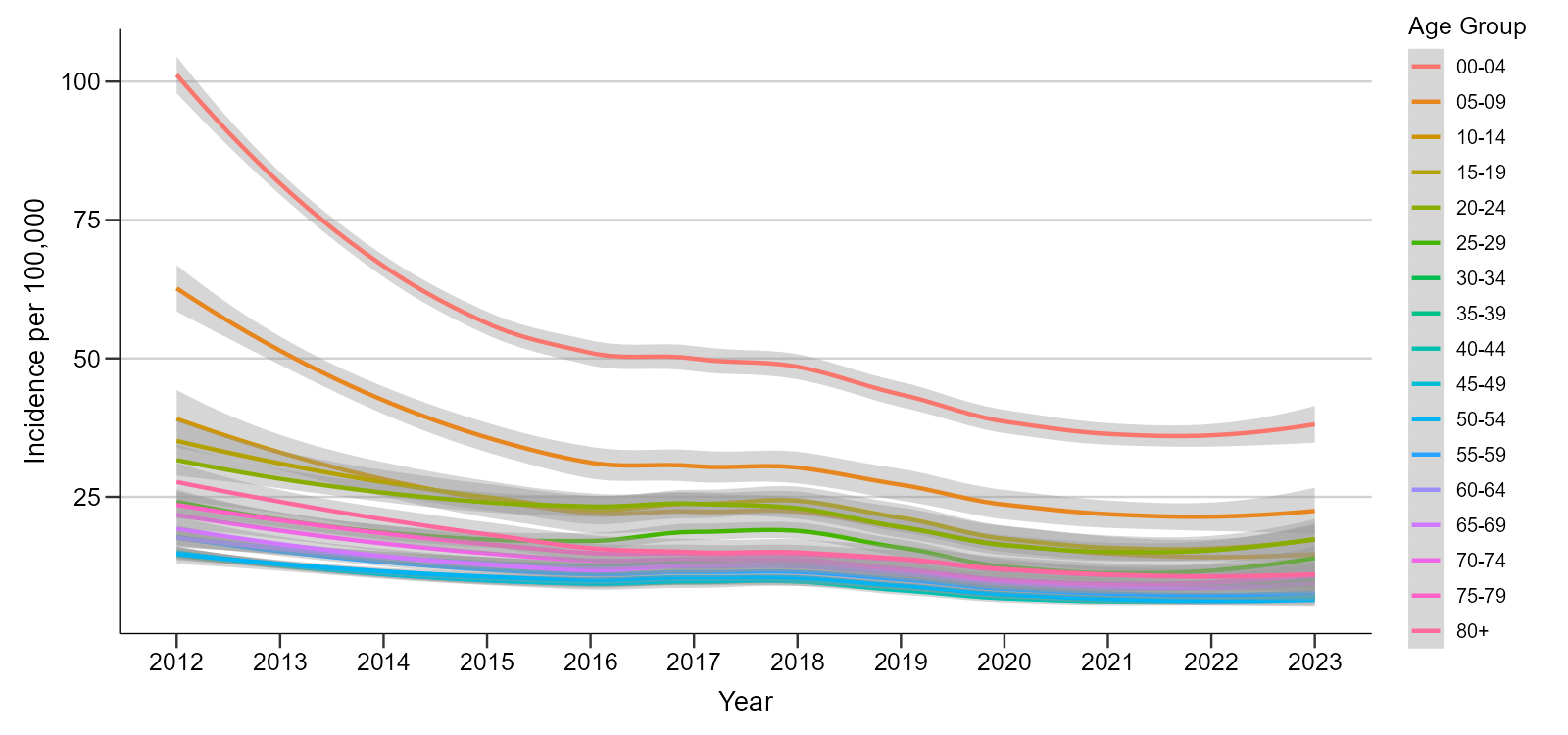


Figure S2. Salmonellosis incidence per 100,000 population by age group from 2012–2023 in Germany


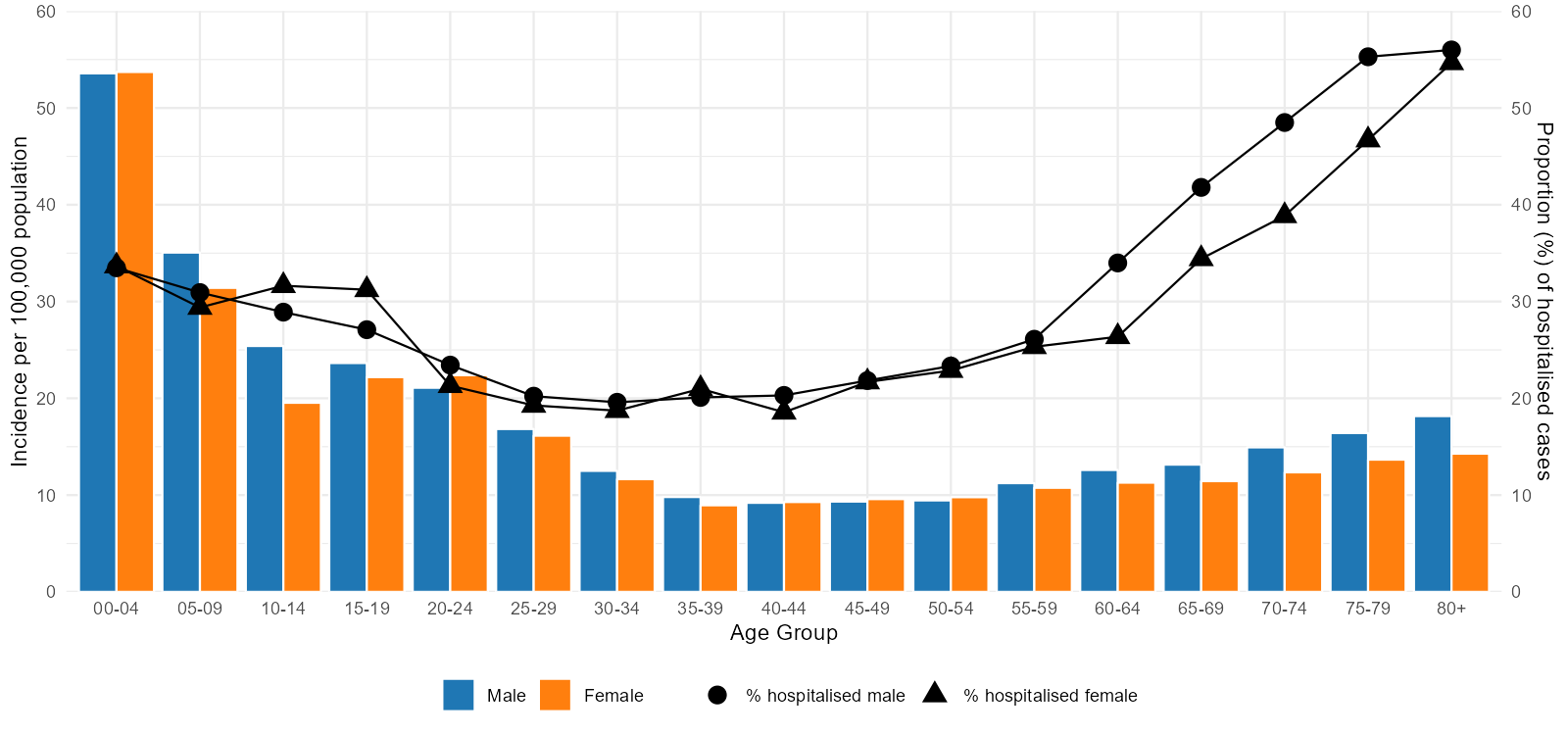


Figure S3. Salmonellosis incidence per 100,000 population by age and gender with proportion of hospitalised cases in Germany from 2012–2023


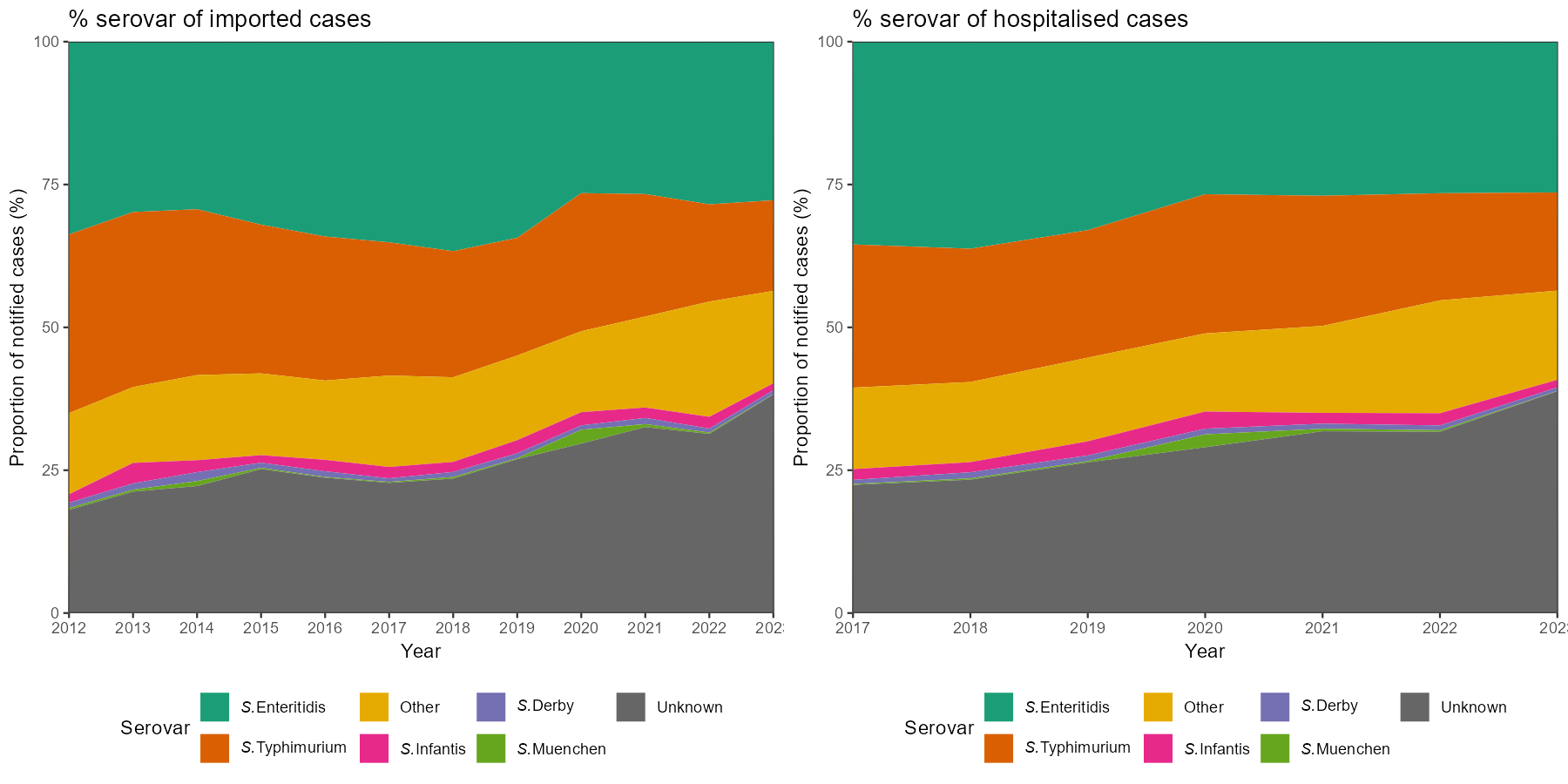


Figure S4 Serovar distribution and trend of imported salmonellosis cases (2012-2023 in Germany) and hospitalised salmonellosis cases (2017–2023 in Germany).
